# The Evolution of Robust Development and Homeostasis in Artificial Organisms

**DOI:** 10.1371/journal.pcbi.1000030

**Published:** 2008-03-28

**Authors:** David Basanta, Mark Miodownik, Buzz Baum

**Affiliations:** 1 Materials Research Group, Engineering Division, King's College London, London, United Kingdom; 2 Laboratory for Molecular Cell Biology, University College London, London, United Kingdom; 3 University College London Branch of the Ludwig Institute for Cancer Research, London, United Kingdom; 4 Zentrum für Informationsdienste und Hochleistungsrechnen, Technische Universität Dresden, Germany; University of Auckland, New Zealand

## Abstract

During embryogenesis, multicellular animals are shaped via cell proliferation, cell rearrangement, and apoptosis. At the end of development, tissue architecture is then maintained through balanced rates of cell proliferation and loss. Here, we take an in silico approach to look for generic systems features of morphogenesis in multicellular animals that arise as a consequence of the evolution of development. Using artificial evolution, we evolved cellular automata-based digital organisms that have distinct embryonic and homeostatic phases of development. Although these evolved organisms use a variety of strategies to maintain their form over time, organisms of different types were all found to rapidly recover from environmental damage in the form of wounds. This regenerative response was most robust in an organism with a stratified tissue-like architecture. An evolutionary analysis revealed that evolution itself contributed to the ability of this organism to maintain its form in the face of genetic and environmental perturbation, confirming the results of previous studies. In addition, the exceptional robustness of this organism to surface injury was found to result from an upward flux of cells, driven in part by cell divisions with a stable niche at the tissue base. Given the general nature of the model, our results lead us to suggest that many of the robust systems properties observed in real organisms, including scar-free wound-healing in well-protected embryos and the layered tissue architecture of regenerating epithelial tissues, may be by-products of the evolution of morphogenesis, rather than the direct result of selection.

## Author Summary

During development, multicellular animals are shaped by cell proliferation, cell rearrangement, and cell death to generate an adult whose form is maintained over time. Disruption of this finely balanced state can have devastating consequences, including aging, psoriasis, and cancer. Typically, however, development is robust, so that animals achieve the same final form even when challenged by environmental damage such as wounding. To see how morphogenetic robustness arises, we have taken an in silico approach to evolve digital organisms that exhibit distinct phases of growth and homeostasis. During the homeostasis period, organisms were found to use a variety of strategies to maintain their form. Remarkably, however, all recovered from severe wounds, despite having evolved in the absence of selection pressure to do so. This ability to regenerate was most striking in an organism with a tissue-like architecture, where it was enhanced by a directional flux of cells that drives tissue turnover. This identifies a stratified architecture, like that seen in human skin and gut, as an evolutionarily accessible and robust form of tissue organisation, and suggests that wound-healing may be a general feature of evolved morphogenetic systems. Both may therefore contribute to homeostasis, wound-healing, and regeneration in real animals.

## Introduction

During development, a mature multicellular animal is generated from a single cell through proliferation, apoptosis and cell rearrangement [1]. Upon reaching adulthood, animals are then able to maintain their form by finely balancing the rates of cell division and cell death. It is important that this unfolding developmental programme be reproducible. In addition, in the real world development and homeostasis must also be robust [2] to mutation [3], to noise internal to the system [4],[5] and to environmental perturbation [6]. This has been verified in experiments, where animals have been shown to recover from profound defects, that include a disruption of normal patterning [3],[7],[8] and severe wounds [9]. Surprisingly, however, several experiments suggest that this capacity to tolerate and to recover from perturbations does not correlate with the expected likelihood of encountering environmental damage. For example, well-protected embryos have been shown to repair wounds better than their mature adult counterparts, without mounting an inflammatory response [6],[10],[11]. Moreover, in several instances, wound-healing has been shown to recapitulate morphogenesis [12]–[14]. It therefore remains an open question how organisms are able to survive these different types of stresses. To help address this question, our aim here was to explore the role of evolution in the generation of morphogenetic robustness, and to study the generic features of homeostasis in complex evolved systems.

A large number of experimental approaches have been used to identify the molecular and cellular processes underlying development and homeostasis in different model biological systems [1],[15],[16]. However, given the length of time over which evolution shapes tissues and organisms, an experimental analysis of the evolution of development and homeostasis in a multicellular animal remains, for the moment, out of reach (although see [17]). To approach this problem, and to begin exploring the generic systems features associated with the evolution of multicellular animal development, we therefore chose to take an artificial life approach [18]; using cellular-automata based digital organisms as subjects for an evolutionary analysis. Although abstractions, such systems usefully recapitulate many aspects of real development [19]. Moreover, this follows a long tradition of research in which cellular automata-based digital organisms are used as experimentally tractable model systems in which to study a variety of problems in evolution and development development [20],[21]. In particular they have been used to the scalability and wound-healing abilities of developmental systems. Miller, for example, showed that evolved cellular automata capable of growing a complex pattern (the French Flag) were robust to a wide variety of environmental perturbations [22], a result that was confirmed in cellular automata guided by a different rule set [23]. Inspired by this work, here we use a developmental CA that we have previously shown to be evolvable [24],[25] to study the role of evolution in the generation of robust developmental patterning and homeostasis.

## Results

**Figure 1:**
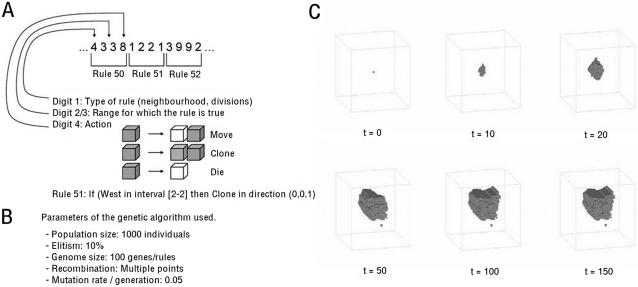
Evolving multicellular digital organisms. (A) The genomes of digital organisms used in this study are made up of 100 rules. 4 numbers define each rule. Digit 1 determines whether the rule is contingent on space (local neighborhood in 3D space) or time (number of divisions). Digits 2 and 3 define the minimum/maximum range of action of each rule (number of local neighbours or interval), and Digit 4 defines the type of action or anti-action to be implemented (to clone a new cell in an adjacent location in the Moore neighbourhood, to move to a neighbouring cell, or to die). An example of a rule is given in full. (B) A genetic algorithm directs organism evolution. (C) The genome of each organism guides its development from a single cell. After allowing for an initial period of growth, organisms are selected that exhibit morphological homeostasis for a period of 100 time-steps (at t = 50, t = 100 and t = 150). Selection is also used to favour organisms that have a surface volume ratio of 0.8 which do not cross the boundary of the 50 by 50 matrix, and to select against fragmented organisms.

As model systems for this study of morphological development and homeostasis we chose 3D cellular automata (CA), whose development is guided by a linear rule-set or ‘genome’ [24],[26]. This consists of 100 rules or ‘genes’ (see Methods), each of which is defined by four integers (Figure 1A and Table S1). One integer specifies an action, to divide (to generate two daughter cells), to move, to die, or to oppose one of these actions. In the case of cell movement or cell cloning events, this integer also dictates the direction in which the action is implemented. The other three integers determine the conditions under which the associated action will be triggered, based upon developmental time, a cell division count, or the number/position of neighbouring cells that each cell directly contacts. This yields (Table S1) a total of 36,842 possible rules or genes, making each organism effectively unique (1 in 100^36,842^). The set of 100 genes that defines each organism constitutes a deterministic developmental programme, which guides the action of every cell in the CA-based organism at each time step, calculated on a majority-win basis [25]. Because cells have their own distinct history and environment, individual cells in each organism follow their own developmental path, despite their having identical genomes. Taking togther, the result is an evolvable CA capable of generating complex three-dimensional forms (see Figure 1C, Methods, [25],[26]), a pre-requisite for this analysis.

With these rules guiding digital organism development in place, a genetic algorithm was then used to select for organisms that exhibit homeostasis (Figure 1B and 1C) [24],[25]. In each case, development was initiated from a single cell. Organisms were allowed 50 time-steps in which to grow, after which we selected for individuals that are best able to maintain their form over a period of 100 time-steps [25],[27]. This homeostatic phase was operationally defined as a minimal change in organismal shape between time-steps 50, 100 and 150, as measured using two simple algorithms, a ‘2-point correlation’ [28] and a lineal path function [29]. From an initial set of 1000 organisms with random genomes, a tournament process was used to select the fittest [26]. These organisms were subjected to a round of mutation and recombination to generate the next generation of 1000. This process of mutation, development and selection was then reiterated for 30 generations, and the process of evolution repeated 36 times to generate a zoo of evolved CAs, which we hoped would include individuals that exhibit distinct developmental phases of growth followed by homeostasis. Such behaviour could of course arise by chance. To assess the likelihood of this occurring, we used the GA to analyse the fitness of a control set of 1000 individuals generated at random (Figure S1A). Although one of these had a modest degree of fitness, the 999 other individuals had negligible fitness scores. Taking this further, we generated movies to visualise the development of each successful individual identified in generation 1 of each run. It was clear from this analysis that there was not a single individual out of a random population of 36,000 that exhibited the desired behaviour-growth followed by homeostasis (as defined in the Methods section). By contrast, the fittest individual in 7 of the 36 (19%) evolutionary runs carried out over 30 generations exhibited clearly defined phases of growth and homeostasis. This analysis confirms that the GA is evolving the CA and verifies that, in the absence of evolution, the likelihood of a homeostatic individual arising by chance is very small.

**Figure 2:**
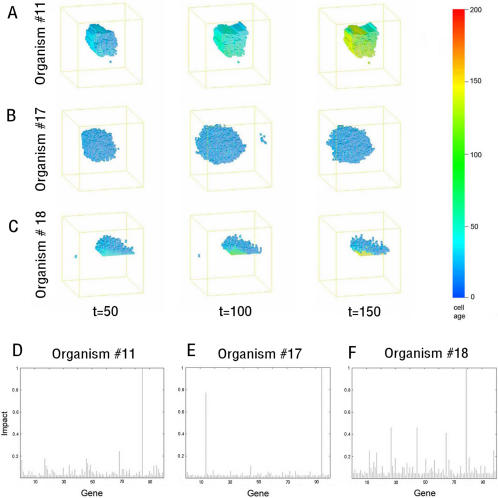
Static and dynamic homeostasis. The pictures are stills of the development of 3 organisms at time steps 50, 100 and 150. Cell age is depicted using a colour key. The organisms exemplify the different types of homeostatic behaviour observed. (A) Organism #11 is static and maintains its form by ossifying, limiting the rate of cell birth, death and movement. (B) Organism #17 is dynamic and maintains an evenly balanced but high rate of cell birth and cell death. (C) In organism #18, dynamic and static regions coexist, so that cells are born at its base and die some time later in the upper regions. This generates a visible gradient of cell ages from lower (old-red) to higher (young-blue) planes. (D–F) Graphs show the impact of mutating individual genes in the genome of organisms #11(D), #17 (E) and #18 (F) on homeostasis. Genes with the highest impact on homeostasis (genes #14 and #94 in organism #17 and gene #80 in organism #18) all encode regulators of cell death (see Videos S2B and S2C).The impact of each mutation was calculated using a combination of 2-point correlation and lineal path analysis between time-steps 50, 100 and 150.

Within this set of evolved homeostatic organisms, two general types of behaviour were used to maintain form during the period from time-step 50 to 150: stasis and dynamic equilibrium. These differences in strategy were clearly visible when cells in each organism were colour-coded according to age (compare Figures 2A, 2B, and 2C). In organism #11, the early period of growth was followed by a period of stasis as the organism takes on a fixed form (Figure 2A) and the cells age rather than die. This resembles morphogenesis in Ecdysoa, such as D. melanogaster and C. elegans, where the majority of cells terminally differentiate upon the completion of development. In contrast, ‘dynamic’ organisms such as #17 achieve homeostasis by maintaining an active balance of cell birth and death (Figure 2B). The remaining 4 homeostatic digital organisms exhibited behaviour somewhere between these two extremes, as exemplified by organism #18 (Figure 2C and Video S3). These were all asymmetric, with a relatively stable domain and a spatially distinct domain characterised by active cell turnover.

To identify the rule-based mechanisms involved in maintaining homeostasis, digital organisms of each type were subjected to a systematic genetic loss of function analysis (Figure 2D, 2E, and 2F). Each gene was removed in turn, and a 2-point correlation and a lineal path function were used once more to measure resultant changes in form. This identified a small number of genes that play a disproportionately large role in the maintenance of homeostasis in each case. In organisms that exhibit balanced cell birth and death, the critical genes involved were all found to have an identical function in promoting cell death during a fixed period of time (genes #14 and #94 in organism #17 and gene #80 in organism #18, Figure 2D, 2E, and 2F). We also noted these genes were over-represented in the genomes of homeostatic organisms compared to random (data not shown). The loss of any one of these genes compromised programmed cell death, causing disorganised growth like that seen in cancer (Videos S7 and S8). A similar overgrowth phenotype is seen when the critical gene #85 is mutated in organism #11 (data not shown). Thus, deregulated growth appears to be a major source of vulnerability in homeostatic digital organisms, as it is in real organisms [30]. In spite of this, most individual genes play a minor role in the maintenance of homeostasis (Figure 2D, 2E, and 2F), reflecting a significant level of functional redundancy in evolved organisms [2],[31].

**Figure 3:**
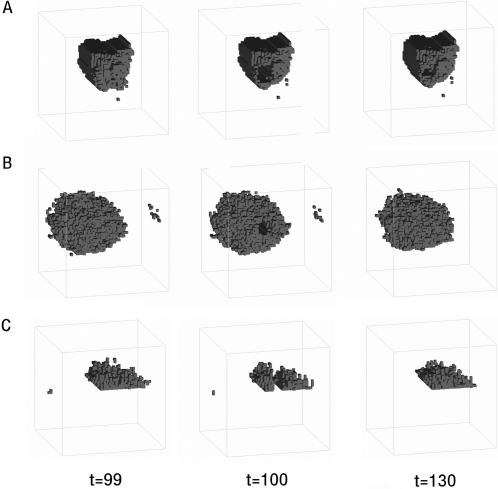
The ability of homeostatic organisms (A) #11, (B) #17, and (C) #18 to withstand environmental perturbation was tested by inducing wounds at time step 100. The recovery process was then followed over time.

Having tested the response of evolved organisms to genetic perturbation, we next tested their ability to maintain morphological homeostasis in the face of an environmental challenge. For this analysis, each organism was subjected to a systematic series of ‘gun-shot’ wounds in which ∼5% or more of the total cell population was removed at time-step 100 (Figure 3). As before, a 2-point correlation and a lineal path function were used to quantify any ensuing repair response. Remarkably, organisms of each type (dynamic, static or asymmetric) were able to heal wounds encompassing hundreds of cells within ∼30 time-steps (Figure 3A, 3B, and 3C and Videos S4, S5, and S6). This wound-healing response was most striking in the case of organism #11 (Figure 3A and Video S4), which during normal development undergoes a period of steady growth, followed by a period of stasis in which cell division and cell death rates fall to zero. Upon wounding, however, this organism mounted an effective repair-response; closing wounds to achieve a good restoration of the organism's original static form. Although normally static, this organism is therefore poised ready to respond appropriately to a variety of environmental insults and, as such, has achieved a relatively sophisticated form of homeostasis.

**Figure 4:**
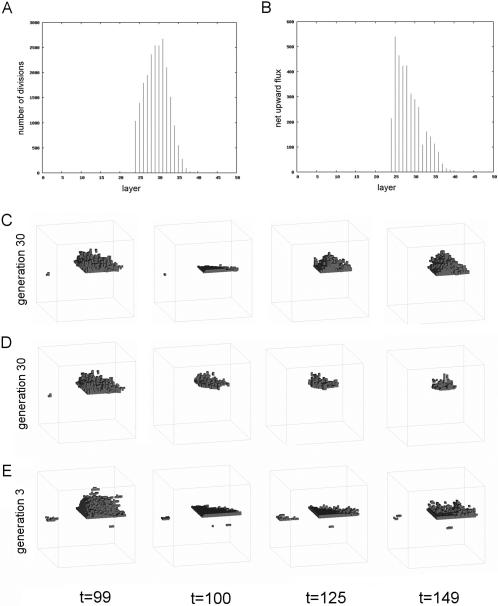
The development of a robust stratified tissue architecture. A series of analyses were used to examine regional cellular behaviour in organism #18. (A) The rates of cell proliferation and (B) the direction of cell movement in organism #18 were calculated layer by layer and are depicted as a bar chart. Organism #18 was then wounded by removal of X cells from (C) the top part or (D) the bottom part, and the response followed over time as indicated. Ancestors of organism #18 were then traced back in evolutionary time using a BLAST-type algorithm. The earliest ancestor with a similar genotype and phenotype was identified in generation 3, after two rounds of selection (see Table S1). (E) This organism was wounded as above, but is unable to heal.

In order to study the evolution of homeostasis, however, we focused our attention on organism #18. This organism was chosen as a subject for further analysis because its layered architecture serves as a useful point of reference for the systematic analysis of regional cellular behaviour and for the generation of equivalent wounds amongst morphological variants. Importantly, these features are a pre-requisite for the quantitative analysis of the evolution of homeostasis and wound-healing. We began the analysis of homeostasis in organism #18 by developing computational methods to visualise the distribution of cell divisions within its different layers (moving from bottom to top) (Figure 4A). During the homeostatic phase, a graded pattern of cell birth was observed along this axis (from bottom to top), with most cell divisions occurring in the central portion and fewer divisions occurring within its stable base or within its dynamic upper layer. In addition, a net flux of cells was observed moving from the bottom to the top of this organism (Figure 4B).

**Figure 5:**
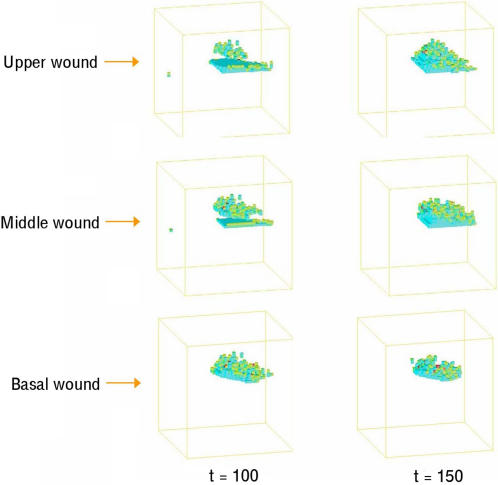
The recovery of organism #18 at generation 30 is shown following wounding at time-step 100 after the removal of a plane of cells at 3 different heights. The colours denote the action being implemented: red denotes cell death and yellow a cell cloning.

Next, we used systematic wounding as a tool to test whether the observed cell behaviour translates into functional differences between different layers in this organism (Figure 4C, 4D, and Figure 5). Following the removal of several upper planes, lost cells were rapidly replenished from below. As a result, this organism was able to rapidly recover from surface wounds that eliminate the majority of its total cell mass (Figure 4C). Similar healing was observed after the removal of a central cell layer (Figure 5). By contrast, the elimination of cells from the stable niche at the base of the organism resulted in a progressive loss of tissue (Figure 4D) and, ultimately, to the organism's disappearance or ‘death’ (data not shown). These data confirm the impression gained by visual inspection (Figure 2C), that a directional flux of cells resulting from cell divisions with the stable ‘stem cell niche’ at the base of this organism drives the turnover of this organisms' upper layers, enabling it to regenerate its form following surface damage.

**Figure 6:**
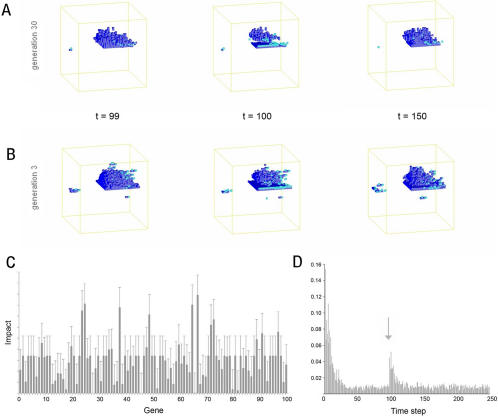
Quantitative analysis of the evolved wound-healing response. (A, B) For this analysis, a wound was generated by removing a slice with thickness equal to five voxels from a central layer in organism #18 at time-step 100. (A) Healing is almost complete after 30 time-steps in the evolved organism at generation 30, whilst the wound remains open at the equivalent time-step in the ancestral organism. In both cases, gene 67 is activated at the wound margin following wounding (highlighted in light blue). (C) A genetic analysis of wound healing in organism #18 was carried out. Each gene was eliminated in turn and the average impact on healing determined using a combination of 2-point correlation and lineal path analysis. 3 lateral wounds (removal of planes at different positions in the X axis at time-step 100) were generated, the average and standard deviation calculated, and the graph normalized to ensure that defects in homeostasis do not confound the analysis. Gene 67 (if (west in interval [7-7] then clone in dir (-1,0,0)), has the greatest relative impact. (D) The proportion of cells in which gene 67 is active is depicted for each time-step during the wounding experiment shown in (A) (see Video S5A). The gene is activated by the morphological changes that accompany wounding (see Video S9) and plays a role in wound-healing (see Video S10).

**Figure 7:**
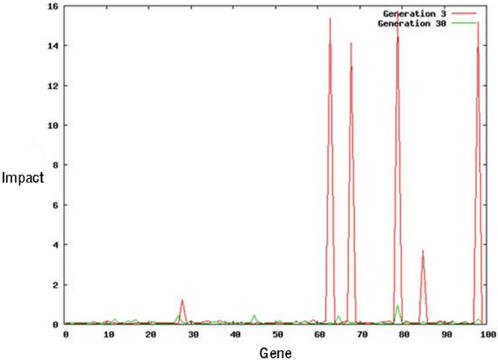
The impact of single gene mutations on homeostasis in organism #18 at generations 3 (red) and 30 (green) are compared. The average impact of a mutation on homeostasis was 0.74 +/− 2.99 at generation 3, and 0.09, +/− 0.12 at generation 30.

We then systematically removed each of this organism's rules in turn to identify the genes involved in the wound-healing response. To do this, 100 single mutant gene variants of organism #18 were generated, each of which was wounded by the removal of a central plane of cells at time-step 100 (Figure 4A). In each case, the extent of recovery over the next 50 time-steps was assessed as a quantitative measure of the effect of each gene on the wound-healing response. It was clear from the spread of phenotypic scores that many genes play a minor role in this process. However, a few genes stood out as having a relatively important role in wound-repair, whilst playing little role during normal development (Figure 4C). We focused our attention on one of these, gene #67 (corresponding to: if (west in interval [7-7]) then clone in dir (−1,0,0)). By visualizing gene activity in time and space during a wound-healing response (Video S11), we were able to show that gene #67 is induced at the wound-margin immediately after wounding (Figure 6A and 6D). Once activated, gene #67 then promotes local cell proliferation in one direction (clone in direction (−1,0,0)) along the axis of the plane. In this way, gene #67 promotes helps restore the organism's original form following wounding. This is the case, even though it plays no role in the normal development of organism #18 in the absence of perturbation (Figure 2F and Figure 7).

**Figure 8:**
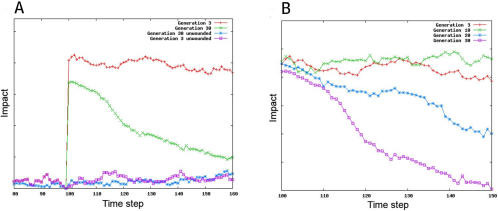
The recovery of organism #18 is depicted following wounding in the lateral plane (A) at generations 3 and 30 (see Videos S11 and S12 for the organism at generation 3 with and without gene 67) and (B) at generations 3, 10, 20 and 30 in the evolutionary process. Wounds (5 voxels in width at position 25) were induced at time-step 100 and the recovery of form followed using a 2 point correlation and lineal path analysis over time. The graphs were normalized for wound size.

**Figure 9:**
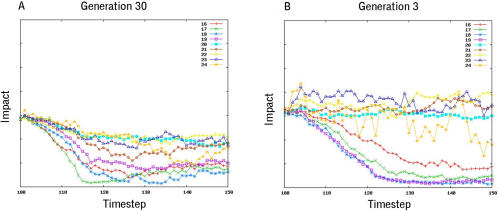
The recovery of organism #18 at (A) generation 30 and (B) generation 3 is shown following wounding at various positions in the Y axis at time-step 100 (see Video S1F) and in (B). The impact and recovery were followed using a 2 point correlation and lineal path analysis over time. Graphs were normalized for wound size.

To explore the role of evolution in the establishment of this repair response, we developed a BLAST-like algorithm [32] which compares genomes in each generation, and then uses homology to trace the lineages of individual organisms back in evolutionary time. This approach identified an ancestor of organism #18 at generation 3 that was similar to its descendent at generation 30 in both its morphology and behaviour (Figure 6B). Interestingly, this ancestral organism was found to carry a copy of gene #67 (Table S2), which plays a small, but measurable role in its development (Figure 7 and data not shown). Taking advantage of this conservation of form during the evolution of organism #18, we were able to generate equivalent wounds carry out a comparative analysis to determine the effect of evolution on the repair response. When compared to its descendent, the ancestral form of organism #18 failed to mount a rapid recovery when faced with a series of systematic wounds (compare Figures 4C and 4E, 6A and 6B, Figures 8 and 9, and Videos S13 and S14). Moreover, by looking at its descendents, this capacity to heal a wound was found to gradually improve with evolutionary time (Figure 8). These data show that, in this case, reiterative rounds of mutation, recombination and selection have led to a steady increase in homeostatic robustness, as measured by the ability to withstand environmental perturbation. This is the case even though wound-healing itself did not form part of the selection criteria used in the genetic algorithm.

We then carried out a similar analysis to establish how evolution affects the robustness of organisms in the face of mutation (Figure 7). To accurately assess the effects of genetic perturbation, we calculated the impact of removing of each of the 100 genes in turn on organismal form at generation 3 and generation 30 (measured using 2 point correlation and a lineal path function). This revealed that the evolved organism #18 at generation 30 is 8-fold more robust to systematic gene deletions than its ancestor, (the average difference in form was 0.74 (std dev. 2.99) for generation 3, and 0.09 (std dev. 0.12) for generation 30). Moreover, a similar result was obtained when we discarded the 4 genes with the highest impact from this analysis (0.1381 (std dev. 0.3886) for generation 3 compared with 0.0437 (std dev. 0.0195) for generation 30). This trend is exemplified by the behaviour of gene #67, which plays a visible role in the control of homeostasis in the ancestral organism, but which is phenotypically silent in generation 30, in the absence of perturbation (Figure 2F). These data show that the increase in fitness seen during the evolution of this organism (Figure S1B and S1C) is accompanied by an increase in its robustness in response to both genetic and environmental perturbation.

## Discussion

Our analysis using artificial multicellular organisms has revealed several important general features of homeostatic systems that contribute to wound-healing and tissue regeneration. First, it shows that a robust wound-healing response arises as an indirect consequence of morphological evolution. This feature of evolved developmental systems has been observed before, firstly by Miller [22],[33], who used CA to model a growth of a pattern, in this case the French Flag as conceived by Lewis Wolpert [1]. This CA used cell-cell interactions and the diffusion of morphogen-like information to construct and pattern a French Flag of defined size. In doing so, Miller showed that this model has the remarkable property of being robust in the face of environmental perturbations, as a by-product of evolution Miller, 2003 #54; Liu, 2005 #60]. More recently, these findings have been taken further by Federici and Downing [23], who approached the problem using a different developmental model based on a neural network that included the ability of cells to emit and detect chemicals, yielding similar results.

Building on this work, in this study we have used a simple GA (that excludes the use of morphogens to induce action at a distance), to evolve digital 3D multicellular organisms that exhibit a growth phase followed by homeostasis, without defining a specific form they should adopt. This helps to generalise the results of Miller, Federici and Downing and identifies a variety of mechanisms that can undelie morphological homeostasis. In addition, by focusing on one organism, we were able to undertake a detailed mechanistic analysis to reveal important features of these evolved developmental CA systems that contribute to their morphological robustness.

We began this mechanistic analysis of the wound-repair response by identifying the key genes involved. One of the most important proved to be gene #67. Upon wounding, this gene was found to be activated at the wound margin, where it promotes cell division, helping to restore the organism's original form (Figure 4 and Figure 9). Interestingly, although this gene is dispensable for normal development of organism #18 at generation 30, earlier in the evolutionary process, at genearation 3, gene #67 was found to facilitate normal homeostatic development. Thus, gene #67 follows the overall trend in which individual genes exhibit an increasing functional redundancy (a reduced impact on development) during the course of evolution (Figure 7). This increasing genetic redundancy is a common feature of evolved systems [2],[31], and suggests a link between evolution, functional redundancy and system robustness. Although this may seem puzzling, genes like gene #67, which appear largely phenotypically silent, can be selected for during evolution if they promote phenotypic stability in the face of the genetic noise that necessarily accompanies rounds of mutation, recombination and selection. In doing so, they help to ensure that incremental changes in the genetic makeup of the evolving organism do not translate into catastrophic changes in form. Based upon this analysis, we hypothesise that many of the genes identified as important for wound-healing in real embryos [9],[13] may have evolved in a similar way to buffer developmental patterning from genetic noise.

This study also reveals an important role for tissue dynamics in the ability of an organism to withstand environmental perturbation. Although all the homeostatic organisms tested displayed a remarkable capacity to heal a wound, organism #18 was unique in being able to recover from profound surface wounds that removed the majority of its total cells, which was related to its capacity for self-renewal. In this organism, cells born in a stable niche at its base establish a directional flow of cells towards the organism's upper surface. This protects the organism from damage, whilst leaving it vulnerable to wounds that affect the stable niche, and to mutations that deregulate cell death (e.g. loss of gene #80). Given the general nature of our model, it is not surprising to find similar systems properties in real tissues constructed using an equivalent architecture. This is evident in surface epithelial tissues, such as the human skin or gut, which continue functioning throughout the lifespan of the organism in the face of continual damage from the outside. Their ability to maintain a constant form and function over time relies on a small population of stem cells embedded within a ‘niche’ at the tissue base [34]–[36]. At each division, these stem cells self-renew to generate a daughter stem cell that remains within the niche, together with a second daughter cell that divides multiple times to generate an overlying population of transit amplifying cells. These rapidly dividing cells then differentiate as they move up through the tissue, giving the stratified tissue its dynamic form; as seen in organism #18 (Figure 4A and 4B). Because of this, like organism #18, stratified epithelia are relatively robust to surface damage, but are vulnerable to the loss of the stem cell niche and to genetic defects in the stem cell compartment; defects, which are linked to aging and cancer, respectively [37]. This appears to be a simple, evolutionary accessible form of homeostasis, since this type of globally polarised cell behaviour was independently evolved in 4 out of 7 homeostatic organisms.

In conclusion, by studying cellular automata-based organisms we have been able to follow the evolution of generic systems features that contribute to their capacity to maintain their form and to recover from wounding, something that is impossible in real organisms. In this way we have shown that wound-healing can arise as an indirect consequence of evolution itself and is most effective in organisms that have a dynamic, self-renewing stratified tissue organisation, like that seen in the human skin and gut.

## Methods

In this section, we describe the development of the rules used to guide the development (the CA) and evolution (the GA) of cellular automata-based digital organisms used in this study. Out goal was to keep this model as simple as possible. To achieve this, i) we began each developmental run with a single cell, ii) we limited the number of choices available to cells at each time-step to cell division, cell movement, or cell death (and excluded action at a distance), iii) we limited cell communication to direct cell-cell interactions, and iv) we assumed that each cell in the organism implements the same deterministic program. Then, beginning with a random set of organisms, each carrying a rule-set of 100 genes, we selected for organisms with a stable form over 100 timesteps, through 30 rounds of mutation, recombination and selection. This identified a set of successful homeostatic organisms. The specifics of how this was implemented are laid out in more detail below. The code itself is available from our web site: https://sourceforge.net/projects/evhomeostasis/.

### Cellular Automata

CA have frequently been used in biomedical research to model a variety of biological processes [20]. The CA employed here differs from many other models [38],[39] in that it the rule-set been designed to be evolvable [25]. Cellular Automata (CA) develop in a simple cubic 3D lattice with 50×50×50 sites or voxels, each of which can be in one of two states; either occupied by a cell or empty. Cell behaviour is determined by a set of 100 rules (the rule-set or genome) that is inherited by all cells in a given CA. This has the following structure:
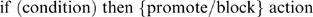
Rules are defined by a string of 4 integer numbers, which specify the conditions under which a rule is active, together with the action it implements. Rules are contingent either on internal factors, i.e. the number of divisions that a cell has undergone or the total number of time-steps since the beginning of the simulation, or on environmental conditions. This is defined as occupancy of the 26 adjacent sites in a cell's local 3D neighborhood (9 below, 9 above and 8 in the plane). Once the designated precondition is satisfied, the rule is activated.

The initial state of the system in each simulation is a single occupied cell in an empty lattice. At each time step, the current state of each cell is evaluated in order to determine which action, if any, it will perform. A cell then implements one of three actions: to move to a neighbouring location, to divide (creating a copy in a neighbouring location), or to die (see Figure 1A); or it implements one of the equivalent anti-actions. Because at any one time a large number of rules may have their preconditions satisfied, a conflict resolution system is used to decide the course of action a given cell will take:

1. Get list of rules whose precondition is true.
2. For positive acting rules: increase the counter associated with the action.
3. For negative acting rules, decrease the counter associated with the action.
4. Pick the action with the higher counter.
5. If counter of selected action is higher than a given threshold, execute action.

Because more than one rule may be applicable in any cell at any given time, actions are complex decisions. As a result, mutations do not carry the same weight as they do in conventional CA, so that small changes in the genome translate into comparatively small changes in the phenotype. Crucially, this makes the CA model highly evolvable, as compared to other CA models. In addition, this system ensures that the behaviour of each cell is unique and CA behaviour rich, as a vast set of possible 3D forms is explored.

### The Evolutionary Algorithm

Genetic algorithms (GA) are a class of optimisation algorithms that use ideas inspired by Darwinian evolution (survival of the fittest and inheritance with variation) to evolve a population of potential solutions to a problem. In this study a GA was used to search for CA that display homoeostasis. Figure 1C shows the development of an evolved individual. Starting from a single cell, the rules direct the growth of an organism during the first 50 time-steps. Successful individuals like the one illustrated are then capable of maintaining this shape for another 100 more time-steps.

The GA contains populations of rule-sets that are encoded by strings of integer numbers (Figure 1A). In this instance, the starting population of 1000 individuals (each with 100 rules that consist of 4 numbers) is created at random [40]. At each generation, the best 50 rulesets are selected based on their ability to maintain their form and are transmitted unchanged to the next generation. The remaining 950 slots in the next generation are filled using a tournament selection, in which three randomly chosen individuals in the population are pitted against one another. Winners are then subjected to a round of recombination and mutation, in which a two-point crossover operator exchanges a portion of 2 chosen genomes at a random position. Mutations lead to the replacement of rules with another chosen at random, with a probability of 0.05/rule.

The fitness function measures the ability of an individual to: (a) to minimise changes in form that occur between time-steps 50, 100 and 150 (b) to grow into a 3D form in which cells are connected to the same body, (c) to grow to a 3D form with a particular surface to volume ratio (in this instance, between 0.5 and 0.8), and to select against individuals that cross the boundary of the 50×50×50 lattice. These criteria select for large, compact homeostatic organisms, and select against infinite columns. The better the individual is able to fulfil these criteria the higher the fitness value. For (a), a two point correlation and a lineal path function are used to compare organismal form at the different time-steps. Rintoul and Torquato [41] have found that the combination of two point correlation and lineal path function can characterise faithfully a large range of spatial patterns. The lineal path function L (x,x+r) is defined as the probability of finding a line segment with end points at x and x+r that lies entirely in the body of the individual. The lineal path function contains connectedness information along a lineal path and hence reflects some long range information about object form. Cule et al. [27] have found that the probability of finding strings of different sizes that fall entirely within the body provides an efficient measure of the lineal path. To obtain the lineal path function:

- For each voxel that contains a cell in the row or column, a string is placed with one end in that voxel and orthogonal to the row or column.
- A counter is increased when a string falls entirely within the body of the individual
- A distribution is obtained by dividing the average of all successful results by the total number of tests. The distribution has one entry for each string size used.

The two point correlation of a phase in a digitised medium can be interpreted as the probability of finding two points in the same state at different distances. A two point correlation function takes the form given by equation (1):
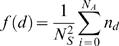
(1)where *d* is the correlation distance, *N_s_* is the total number of occupied cells in the CA, and *n_d_* is the number of alive cells separated by distance *d* from cell *i*.

The different criteria mentioned to compute the adequacy of an individual (volume to surface ratio, degree of connectedness of cells and degree of homoeostasis at time steps 50, 100 and 150) are combined into a single fitness value using a method called Sum of Weighted Global Ratios (SWGR) [42]. Using this scheme the three criteria are normalised using the maximum and minimum values found during the evolution. The three normalised values are then added together to obtain a measure of the fitness of the individual.

### Blast Analysis

The Blast analysis [32] allows the comparison of the genomes of two different individuals and produces as a result the percentage of genes in common as well as the locations in which there are differences. The comparison takes places by aligning the two genomes as used in the genetic algorithm and counting all the places in which there is a match and all those in which there is not.

### Genetic Function Analysis

In order to analyse the contribution of each gene in the maintenance of homeostasis we produced 100 single gene deletion mutants. The effect of each mutation on development was then measured using the two point correlation and lineal path functions to compare the form of the wildtype and of each mutant.

### Wounding

The wounding tool removes all the cells of the digital organism that happen to fall within a given radius of the wound axis (X, Y or Z). In most cases, we applied a systematic series of ‘gun-shot’ wounds at timestep 100 by shifting the wound in increments in X, Y or Z. The recovery was assessed by measuring the morphological differences between the organism immediately prior to wounding and during the course of its recovery.

### Tracking Gene Activity

A gene could be considered active in two difference senses, i) if the preconditions under which the rule can act are met, ii) if the specified gene action is executed. We used the second definition to track gene activity during organism development. For each timestep, we then measure the number of cells that execute a give rule as a proportion of the total number of cells.

## Supporting Information

Figure S1 The chance of homeostastic organisms with a high fitness arising by chance. (A) Fitness scores are shown for 1000 randomly generated organisms. The best individual has achieved homeostasis by generating an infinite column, and is therefore cannot be considered homeostatic. (B) Fitness scores are shown for the best individuals from generation 1 (in each case from 1000 randomly generated organisms). None show distinct growth and homeostatic phases of development. Those that achieved homeotasis did so by remaining very small or by forming inifinite columns that crossed the boundary of the 50×50×50 space. (C) Fitness scores are shown for the best individuals from 30.
(4.18 MB TIF)

Table S1 Genotype into phenotype.
(0.04 MB XLS)

Table S2 Comparison between genomes of related organisms in run 18 at generation 3 and 30.
(0.01 MB PDF)

Video S1 Homeostasis in evolved digital organisms. Movie shows organism #11 as it develops from a single cell and stabilizes their forms at ∼ time-step 50. The organism exemplifies static homeostasis.
(3.30 MB CDR)

Video S2 Homeostasis in evolved digital organisms. Movie shows organism #17 as it develops from a single cell and stabilizes their forms at ∼ time-step 50. The organism exemplifies dynamic homeostasis.
(3.81 MB CDR)

Video S3 Homeostasis in evolved digital organisms. Movie shows organism #18 as it develops from a single cell and stabilizes their forms at ∼ time-step 50. The organism exemplifies mixed homeostasis.
(2.74 MB CDR)

Video S4 Wound-healing in evolved digital organisms. Movie shows organism #11 as it develops from a single cell, and stabilizes their forms at ∼ time-step 50. The organism was subjected to a ‘gun shot’ wound that removes a core of cells of pixels or a 5 voxel width plane of cells in the Y axis. Actions have been colour-coded so that a yellow cell is about to be cloned, a deep blue cell to move, and a red cell to die.
(3.94 MB CDR)

Video S5 Wound-healing in evolved digital organisms. Movie shows organism #17 as it develops from a single cell and stabilizes their forms at ∼ time-step 50. The organism was subjected to a ‘gun shot’ wound that removes a core of cells of pixels or a 5 voxel width plane of cells in the Y axis. Actions have been colour-coded so that a yellow cell is about to be cloned, a deep blue cell to move, and a red cell to die.
(4.81 MB CDR)

Video S6 Wound-healing in evolved digital organisms. Movie shows organism #18 as it develops from a single cell and stabilizes their forms at ∼ time-step 50. The organism was subjected to a ‘gun shot’ wound that removes a core of cells of pixels or a 5 voxel width plane of cells in the Y axis. Actions have been colour-coded so that a yellow cell is about to be cloned, a deep blue cell to move, and a red cell to die.
(3.36 MB CDR)

Video S7 Organism 17 mutant for gene 14 (a).
(3.58 MB CDR)

Video S8 Organism 17 mutant for gene 14 (b).
(3.72 MB CDR)

Video S9 Organism #18 at generation 30 is shown recovering from a lateral wound (5 voxels in width at position 25) with gene 67. The activity of gene 67 is depicted in light blue, showing that gene 67 is induced at the wound margin following wounding.
(2.69 MB CDR)

Video S10 Organism #18 at generation 30 is shown recovering from a lateral wound (5 voxels in width at position 25) without gene 67.
(6.98 MB CDR)

Video S11 The ancestor of organism #18 at generation 3 is shown recovering from a lateral wound (5 voxels in width at position 25).
(2.31 MB CDR)

Video S12 The ancestor of organism #18 at generation 3 is shown recovering from a lateral wound (5 voxels in width at position 25).
(3.26 MB CDR)

Video S13 Movie of homeostatic organism #4 is shown as it develops from a single cell. It contains a mixture of dynamic and static regions.
(6.98 MB CDR)

Video S14 Movie of homeostatic organism #28 is shown as it develops from a single cell. It contains a mixture of dynamic and static regions.
(4.31 MB CDR)
