# Supplementary material for: The Evolution of Robust Development and Homeostasis in Artificial Organisms
Source: PLoS Comput Biol. 2008 Mar 28;4(3):e1000030. doi: 10.1371/journal.pcbi.1000030 (PMC2274883; doi:10.1371/journal.pcbi.1000030)
Supplement: Table S2 — Comparison between genomes of related organisms in run 18 at generation 3 and 30. (0.01 MB PDF) [file pcbi.1000030.s003.pdf]

Table I

Comparison of the ruleset of organism #18 at generations 3 and 30

**Gene #**	**Generation 3 - genome**	**Generation 30 - genome**
1	if (East in interval [9 - 9]) then = Move to (0,1,0)	if (South in interval [3 - 3]) then = Don't move in dir (0,0,1)
2	**if (CellType in interval [2 - 2]) then = Don't clone in dir (0,1,0)**	**if (CellType in interval [2 - 2]) then = Don't clone in dir (0,1,0)**
3	if (East in interval [4 - 4]) then = Clone in dir (0,0,-1)	if (West in interval [3 - 3]) then = Don't die
4	**if (Division in interval [278 - 487]) then = Don't die**	**if (Division in interval [278 - 487]) then = Don't die**
5	**if (Timesteps in interval [134 - 345]) then = Clone in dir (1,0,0)**	**if (Timesteps in interval [134 - 345]) then = Clone in dir (1,0,0)**
6	**if (Division in interval [49 - 484]) then = Move to (0,0,1)**	**if (Division in interval [49 - 484]) then = Move to (0,0,1)**
7	if (Division in interval [373 - 402]) then = Don't clone in dir (1,0,0)	if (South in interval [4 - 4]) then = Clone in dir (-1,0,0)
8	if (North in interval [3 - 3]) then = Don't clone in dir (1,0,0)	if (Up in interval [8 - 8]) then = Don't move in dir (0,1,0)
9	if (Timesteps in interval [220 - 405]) then = Don't clone in dir (1,0,0)	if (East in interval [2 - 2]) then = Clone in dir (0,0,-1)
10	if (Timesteps in interval [346 - 458]) then = Don't move in dir (0,1,0)	if (North in interval [3 - 3]) then = Clone in dir (0,-1,0)
11	if (Down in interval [5 - 5]) then = Move to (1,0,0)	if (CellType in interval [1 - 1]) then = Don't move in dir (-1,0,0)
12	if (East in interval [1 - 1]) then = Move to (-1,0,0)	if (Timesteps in interval [96 - 382]) then = Clone in dir (0,0,-1)
13	if (CellType in interval [2 - 2]) then = Clone in dir (-1,0,0)	if (South in interval [2 - 2]) then = Die
14	if (East in interval [0 - 0]) then = Don't clone in dir (0,0,-1)	if (West in interval [2 - 2]) then = Move to (0,0,1)
15	**if (Down in interval [8 - 8]) then = Don't die**	**if (Down in interval [8 - 8]) then = Don't die**
16	**if (Up in interval [6 - 6]) then = Die**	**if (Up in interval [6 - 6]) then = Die**
17	**if (Down in interval [5 - 5]) then = Move to (0,-1,0)**	**if (Down in interval [5 - 5]) then = Move to (0,-1,0)**
18	**if (North in interval [0 - 0]) then = Don't die**	**if (North in interval [0 - 0]) then = Don't die**
19	**if (Up in interval [0 - 0]) then = Don't clone in dir (0,0,-1)**	**if (Up in interval [0 - 0]) then = Don't clone in dir (0,0,-1)**
20	if (West in interval [5 - 5]) then = Die	if (East in interval [4 - 4]) then = Don't move in dir (0,-1,0)
21	**if (CellType in interval [1 - 1]) then = Move to (1,0,0)**	**if (CellType in interval [1 - 1]) then = Move to (1,0,0)**
22	if (North in interval [1 - 1]) then = Clone in dir (-1,0,0)	if (West in interval [8 - 8]) then = Move to (0,-1,0)
23	**if (CellType in interval [0 - 0]) then = Don't move in dir (0,-1,0)**	**if (CellType in interval [0 - 0]) then = Don't move in dir (0,-1,0)**
24	**if (North in interval [7 - 7]) then = Don't move in dir (0,1,0)**	**if (North in interval [7 - 7]) then = Don't move in dir (0,1,0)**
25	**if (South in interval [9 - 9]) then = Don't die**	**if (South in interval [9 - 9]) then = Don't die**
26	**if (West in interval [5 - 5]) then = Don't die**	**if (West in interval [5 - 5]) then = Don't die**
27	**if (Division in interval [242 - 493]) then = Die**	**if (Division in interval [242 - 493]) then = Die**
28	**if (Down in interval [1 - 1]) then = Clone in dir (0,-1,0)**	**if (Down in interval [1 - 1]) then = Clone in dir (0,-1,0)**
29	**if (Up in interval [0 - 0]) then = Clone in dir (0,0,1)**	**if (Up in interval [0 - 0]) then = Clone in dir (0,0,1)**
30	**if (Timesteps in interval [328 - 421]) then = Clone in dir (0,0,-1)**	**if (Timesteps in interval [328 - 421]) then = Clone in dir (0,0,-1)**
31	**if (North in interval [3 - 3]) then = Die**	**if (North in interval [3 - 3]) then = Die**
32	**if (CellType in interval [1 - 1]) then = Die**	**if (CellType in interval [1 - 1]) then = Die**
33	**if (Division in interval [454 - 484]) then = Don't die**	**if (Division in interval [454 - 484]) then = Don't die**
34	**if (North in interval [1 - 1]) then = Clone in dir (0,0,-1)**	**if (North in interval [1 - 1]) then = Clone in dir (0,0,-1)**
35	**if (North in interval [6 - 6]) then = Clone in dir (0,0,1)**	**if (North in interval [6 - 6]) then = Clone in dir (0,0,1)**
36	**if (North in interval [0 - 0]) then = Clone in dir (0,0,1)**	**if (North in interval [0 - 0]) then = Clone in dir (0,0,1)**
37	**if (Down in interval [4 - 4]) then = Move to (0,0,1)**	**if (Down in interval [4 - 4]) then = Move to (0,0,1)**
38	**if (Up in interval [5 - 5]) then = Clone in dir (0,0,1)**	**if (Up in interval [5 - 5]) then = Clone in dir (0,0,1)**
39	**if (West in interval [5 - 5]) then = Move to (0,1,0)**	**if (West in interval [5 - 5]) then = Move to (0,1,0)**
40	**if (CellType in interval [2 - 2]) then = Don't die**	**if (CellType in interval [2 - 2]) then = Don't die**
41	if (Timesteps in interval [397 - 483]) then = Die	if (Up in interval [0 - 0]) then = Die
42	**if (CellType in interval [0 - 0]) then = Don't clone in dir (1,0,0)**	**if (CellType in interval [0 - 0]) then = Don't clone in dir (1,0,0)**
43	**if (Down in interval [3 - 3]) then = Die**	**if (Down in interval [3 - 3]) then = Die**
44	**if (Division in interval [249 - 302]) then = Move to (0,-1,0)**	**if (Division in interval [249 - 302]) then = Move to (0,-1,0)**
45	**if (Division in interval [247 - 391]) then = Die**	**if (Division in interval [247 - 391]) then = Die**
46	**if (West in interval [0 - 0]) then = Clone in dir (0,-1,0)**	**if (West in interval [0 - 0]) then = Clone in dir (0,-1,0)**
47	if (Down in interval [6 - 6]) then = Don't move in dir (1,0,0)	if (Timesteps in interval [193 - 200]) then = Don't clone in dir (0,0,1)
48	**if (Up in interval [8 - 8]) then = Move to (0,-1,0)**	**if (Up in interval [8 - 8]) then = Move to (0,-1,0)**
49	**if (East in interval [7 - 7]) then = Don't move in dir (0,-1,0)**	**if (East in interval [7 - 7]) then = Don't move in dir (0,-1,0)**
50	**if (Division in interval [59 - 481]) then = Move to (0,0,1)**	**if (Division in interval [59 - 481]) then = Move to (0,0,1)**
51	**if (CellType in interval [0 - 0]) then = Move to (1,0,0)**	**if (CellType in interval [0 - 0]) then = Move to (1,0,0)**
52	**if (South in interval [1 - 1]) then = Don't clone in dir (0,0,1)**	**if (South in interval [1 - 1]) then = Don't clone in dir (0,0,1)**
53	**if (Timesteps in interval [210 - 238]) then = Clone in dir (0,1,0)**	**if (Timesteps in interval [210 - 238]) then = Clone in dir (0,1,0)**
54	**if (Division in interval [420 - 481]) then = Clone in dir (0,0,-1)**	**if (Division in interval [420 - 481]) then = Clone in dir (0,0,-1)**
55	**if (Up in interval [7 - 7]) then = Clone in dir (0,0,-1)**	**if (Up in interval [7 - 7]) then = Clone in dir (0,0,-1)**
56	**if (Down in interval [6 - 6]) then = Don't die**	**if (Down in interval [6 - 6]) then = Don't die**
57	if (Up in interval [9 - 9]) then = Clone in dir (1,0,0)	if (East in interval [7 - 7]) then = Move to (0,-1,0)
58	if (Up in interval [4 - 4]) then = Don't move in dir (1,0,0)	if (CellType in interval [0 - 0]) then = Move to (0,0,-1)
59	if (Timesteps in interval [142 - 334]) then = Don't clone in dir (0,1,0)	if (West in interval [6 - 6]) then = Don't clone in dir (0,-1,0)
60	if (West in interval [4 - 4]) then = Die	if (Up in interval [7 - 7]) then = Don't clone in dir (0,0,1)
61	if (Division in interval [359 - 378]) then = Don't die	if (Down in interval [4 - 4]) then = Die
62	**if (Division in interval [215 - 422]) then = Move to (0,0,-1)**	**if (Division in interval [215 - 422]) then = Move to (0,0,-1)**
63	**if (West in interval [3 - 3]) then = Don't clone in dir (0,1,0)**	**if (West in interval [3 - 3]) then = Don't clone in dir (0,1,0)**
64	**if (Division in interval [34 - 270]) then = Die**	**if (Division in interval [34 - 270]) then = Die**
65	if (Timesteps in interval [2 - 116]) then = Clone in dir (0,1,0)	if (Up in interval [8 - 8]) then = Move to (0,0,1)
66	**if (South in interval [2 - 2]) then = Clone in dir (1,0,0)**	**if (South in interval [2 - 2]) then = Clone in dir (1,0,0)**
67	**if (West in interval [7 - 7]) then = Clone in dir (-1,0,0)**	**if (West in interval [7 - 7]) then = Clone in dir (-1,0,0)**
68	**if (West in interval [0 - 0]) then = Don't die**	**if (West in interval [0 - 0]) then = Don't die**
69	**if (Division in interval [41 - 309]) then = Die**	**if (Division in interval [41 - 309]) then = Die**
70	**if (North in interval [7 - 7]) then = Move to (0,1,0)**	**if (North in interval [7 - 7]) then = Move to (0,1,0)**
71	if (CellType in interval [0 - 0]) then = Don't clone in dir (1,0,0)	if (Up in interval [7 - 7]) then = Don't move in dir (0,0,-1)
72	**if (West in interval [5 - 5]) then = Clone in dir (0,0,-1)**	**if (West in interval [5 - 5]) then = Clone in dir (0,0,-1)**
73	if (West in interval [5 - 5]) then = Clone in dir (0,0,-1)	if (South in interval [7 - 7]) then = Don't clone in dir (1,0,0)
74	**if (Timesteps in interval [104 - 179]) then = Don't move in dir (1,0,0)**	**if (Timesteps in interval [104 - 179]) then = Don't move in dir (1,0,0)**
75	if (South in interval [5 - 5]) then = Don't die	if (East in interval [2 - 2]) then = Don't clone in dir (0,1,0)
76	**if (West in interval [4 - 4]) then = Clone in dir (1,0,0)**	**if (West in interval [4 - 4]) then = Clone in dir (1,0,0)**
77	if (Up in interval [6 - 6]) then = Don't move in dir (1,0,0)	if (Timesteps in interval [31 - 256]) then = Clone in dir (0,0,1)
78	**if (East in interval [6 - 6]) then = Don't die**	**if (East in interval [6 - 6]) then = Don't die**
79	**if (CellType in interval [1 - 1]) then = Don't move in dir (0,1,0)**	**if (CellType in interval [1 - 1]) then = Don't move in dir (0,1,0)**
80	**if (Division in interval [23 - 491]) then = Die**	**if (Division in interval [23 - 491]) then = Die**
81	if (East in interval [7 - 7]) then = Clone in dir (0,0,1)	if (Timesteps in interval [356 - 498]) then = Don't die
82	**if (East in interval [5 - 5]) then = Move to (0,1,0)**	**if (East in interval [5 - 5]) then = Move to (0,1,0)**
83	if (Timesteps in interval [23 - 40]) then = Don't clone in dir (1,0,0)	if (Timesteps in interval [303 - 426]) then = Move to (0,0,-1)
84	**if (North in interval [6 - 6]) then = Don't clone in dir (1,0,0)**	**if (North in interval [6 - 6]) then = Don't clone in dir (1,0,0)**
85	**if (Division in interval [338 - 410]) then = Move to (0,1,0)**	**if (Division in interval [338 - 410]) then = Move to (0,1,0)**
86	**if (Up in interval [0 - 0]) then = Die**	**if (Up in interval [0 - 0]) then = Die**
87	**if (Down in interval [0 - 0]) then = Don't die**	**if (Down in interval [0 - 0]) then = Don't die**
88	**if (Timesteps in interval [171 - 488]) then = Clone in dir (0,1,0)**	**if (Timesteps in interval [171 - 488]) then = Clone in dir (0,1,0)**
89	if (CellType in interval [1 - 1]) then = Clone in dir (0,0,1)	if (Division in interval [31 - 164]) then = Clone in dir (1,0,0)
90	**if (North in interval [4 - 4]) then = Move to (1,0,0)**	**if (North in interval [4 - 4]) then = Move to (1,0,0)**
91	if (East in interval [1 - 1]) then = Die	if (East in interval [6 - 6]) then = Clone in dir (0,-1,0)
92	**if (Timesteps in interval [151 - 286]) then = Die**	**if (Timesteps in interval [151 - 286]) then = Die**
93	**if (North in interval [5 - 5]) then = Clone in dir (0,0,-1)**	**if (North in interval [5 - 5]) then = Clone in dir (0,0,-1)**
94	if (South in interval [6 - 6]) then = Don't move in dir (0,-1,0)	if (CellType in interval [0 - 0]) then = Move to (0,1,0)
95	**if (Timesteps in interval [152 - 235]) then = Don't die**	**if (Timesteps in interval [152 - 235]) then = Don't die**
96	if (Down in interval [4 - 4]) then = Don't clone in dir (0,-1,0)	if (Timesteps in interval [201 - 327]) then = Move to (0,1,0)
97	**if (South in interval [3 - 3]) then = Don't move in dir (0,-1,0)**	**if (South in interval [3 - 3]) then = Don't move in dir (0,-1,0)**
98	**if (Up in interval [4 - 4]) then = Don't move in dir (0,0,-1)**	**if (Up in interval [4 - 4]) then = Don't move in dir (0,0,-1)**
99	**if (Division in interval [28 - 300]) then = Die**	**if (Division in interval [28 - 300]) then = Die**
100	if (Down in interval [3 - 3]) then = Move to (1,0,0)	if (North in interval [2 - 2]) then = Don't clone in dir (0,0,1)
